# Targeting PAD4: A Promising Strategy to Combat β-Cell Loss in Type 1 Diabetes

**DOI:** 10.3390/ijms26136113

**Published:** 2025-06-25

**Authors:** Hsu Lin Kang, András Szász, Zsuzsanna Valkusz, Tamás Várkonyi, Anikó Pósa, Krisztina Kupai

**Affiliations:** 1 Department of Oral Biology and Experimental Dental Research, Faculty of Dentistry, University of Szeged, 6703 Szeged, Hungary; 2 Department of Internal Medicine, Albert Szent-Györgyi Medical School, University of Szeged, 6703 Szeged, Hungaryvarkonyi.tamas@med.u-szeged.hu (T.V.)

**Keywords:** STZ, PAD4, Wistar rat

## Abstract

Peptidylarginine deiminase 4 (PAD4) catalyzes protein citrullination, a post-translational modification implicated in type 1 diabetes mellitus (T1DM). This study examined PAD4 expression and activity in the pancreas of streptozotocin (STZ)-induced diabetic Wistar rats. Animals were divided into three groups: (A) STZ-induced diabetic rats (60 mg/kg, i.p.), (B) non-diabetic controls, and (C) diabetic rats treated with Cl-amidine (5 mg/kg), a pan-PAD inhibitor, from week six post-induction. Analyses included PAD4 mRNA and protein expression, citrullinated histone H3 (CitH3), calcium concentration, and neutrophil elastase activity. Diabetic rats exhibited increased PAD4 expression, CitH3 levels, and NETosis markers, alongside reduced pancreatic calcium, suggesting calcium consumption during PAD4 activation. Cl-amidine treatment attenuated NETosis. These results implicate PAD4 in T1DM pathogenesis via NETosis and support the utility of STZ-induced diabetic rats as a model for PAD4-targeted studies. Cl-amidine may represent a promising therapeutic approach to reduce pancreatic inflammation in T1DM.

## 1. Introduction

Peptidylarginine deiminase 4 (PAD4) is a calcium-dependent enzyme that catalyzes the post-translational deimination of arginine residues to citrulline—a process termed citrullination. This modification alters protein structure and function, particularly in nuclear proteins, such as histones, and plays a central role in chromatin remodeling, gene regulation, and immune responses. Dysregulated PAD4 activity has been implicated in several autoimmune and inflammatory diseases, including type 1 diabetes mellitus (T1DM), rheumatoid arthritis, and systemic lupus erythematosus [1,2,3].

PAD4, uniquely localized to the nucleus among the five PAD isoforms, is predominantly expressed in neutrophils. One of its most studied functions is the induction of neutrophil extracellular traps (NETs)—web-like structures composed of decondensed chromatin and neutrophil enzymes. While NETosis plays a beneficial role in antimicrobial defense, its excessive activation contributes to chronic inflammation and autoimmunity [4]. In the context of T1DM, increased PAD4 expression and NET formation have been associated with insulitis and β-cell destruction in both clinical and preclinical studies.

Histone H3 citrullination (CITH3), a PAD4-mediated modification, is widely recognized as a hallmark of NET formation and serves as a surrogate marker of PAD4 activity. Increasing evidence indicates that hyperglycemia upregulates PAD4 in neutrophils and enhances NETosis, thereby exacerbating tissue injury and inflammation in diabetes [5,6,7]. Inhibiting PAD4 using pharmacological agents like Cl-amidine has shown promising results in mouse models, including improved wound healing, reduced inflammatory markers, and dampened NET formation [8].

Although PAD4 has been well-characterized in mice, significantly fewer studies have focused on PAD4 expression and function in rat models of T1DM, particularly in the pancreas. Prior studies have explored PAD4 signaling and Cl-amidine effects in mouse models of streptozotocin (STZ)-induced diabetes [8,9], but comprehensive investigations in rats remain scarce. A limited number of studies have addressed PAD-related NETosis or histone modifications in rat tissues, but without directly evaluating pancreatic PAD4 expression in the context of STZ-induced diabetes or therapeutic inhibition [10]. Therefore, rather than claiming first-ever application, our study aims to extend and contextualize previous findings by systematically examining PAD4 mRNA and protein expression, histone citrullination, calcium levels, and inflammatory cytokines in the pancreas of diabetic rats with and without Cl-amidine treatment.

Species-specific differences between rats and mice are well-established in terms of STZ susceptibility, immune responses, and pharmacokinetics. As highlighted by Furman, rats may offer greater tissue yield and more physiologically relevant models for certain metabolic and inflammatory processes [11]. Thus, while our model is not the first to investigate PAD4 in diabetes, it provides a pancreas-focused, rat-based system that complements prior mouse studies and may enhance translational validity.

Indeed, PAD4 is increasingly recognized as a relevant immunomodulatory target in human T1DM. Recent human studies have shown elevated levels of PAD4 and NET-derived markers (e.g., CITH3, cell-free DNA) in the blood of patients with T1DM [6,12,13,14,15]. These markers correlate with glycemic control and systemic inflammation and may serve as early biomarkers or therapeutic targets. Furthermore, Yang and colleagues proposed PAD4-related signatures as predictors of autoimmune disease activity, adding weight to the idea that PAD4 inhibitors, like Cl-amidine, could offer benefit beyond animal models [7].

While Cl-amidine is widely used as a PAD inhibitor, potential off-target effects have been reported, including the modulation of apoptosis, oxidative stress, and immune cell function [16,17,18]. In diabetic models, Cl-amidine has been shown to reduce hyperglycemia-induced NETosis and inflammation, improve wound healing, and attenuate tissue injury—effects largely attributed to PAD inhibition but potentially involving additional mechanisms [6,19,20]. These pleiotropic effects are particularly relevant in diabetes, where oxidative stress and immune dysregulation are prominent features. Thus, while our study focuses on PAD4-related pathways, the broader actions of Cl-amidine are acknowledged and controlled for using appropriate vehicle groups. This consideration strengthens the interpretation of PAD4-specific contributions to pancreatic inflammation in STZ-induced diabetes.

Taken together, this study seeks to expand the current knowledge by evaluating PAD4 signaling, NET markers, and calcium homeostasis in the diabetic rat pancreas. We also assess the modulatory effect of Cl-amidine in this model, aiming to clarify PAD4’s involvement in pancreatic inflammation and its potential as a therapeutic target in T1DM. Moreover, increased PAD4 and NET markers have been observed in T1DM patients, supporting the clinical relevance of this pathway [21]. Our study aims to explore these mechanisms in a rat model to strengthen the translational link.

## 2. Results

### 2.1. Verifying That STZ Injured the Pancreatic Tissue, Not the Liver, at the SIXTH Week in Exp A

**Figure 1:**
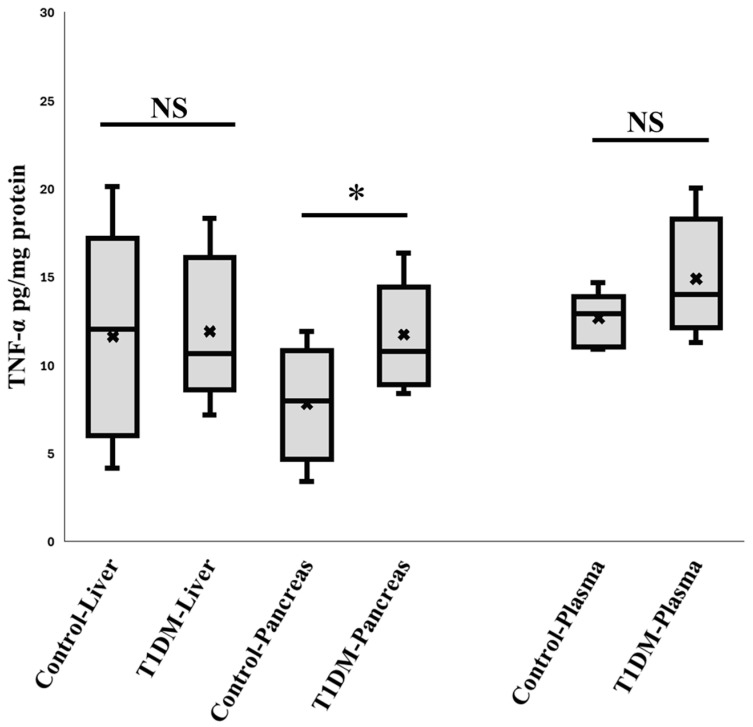
The expression of TNF- α in the plasma, liver, and pancreas. According to the comparison, over the first six weeks, STZ may only damage DNA in the pancreas through GLUT2. Neither the liver nor the other organs were damaged. (n = 10), * *p* < 0.05.

Since STZ enters cells through Glucose transporter 2 (GLUT2) [22], which is abundant in the liver and pancreas, it was first necessary to confirm that STZ harmed β cells in the pancreas rather than the liver. Therefore, liver inflammation may draw granulocytes; however, our findings show no discernible liver inflammation based on TNF-α expression measurements (Figure 1).

### 2.2. Validating the Inflammation in Pancreas During the Sixth Week in Exp A

As demonstrated in Table 1, STZ-induced diabetes was associated with a significant decrease in the anti- inflammatory cytokine IL-10 and an increase in the proinflammatory cytokines CXCL-1, IFN-γ, IL-6, IL-18, and IL-33.

### 2.3. PAD4 mRNA Level in the Pancreas at the Sixth Week in Exp A

**Figure 2:**
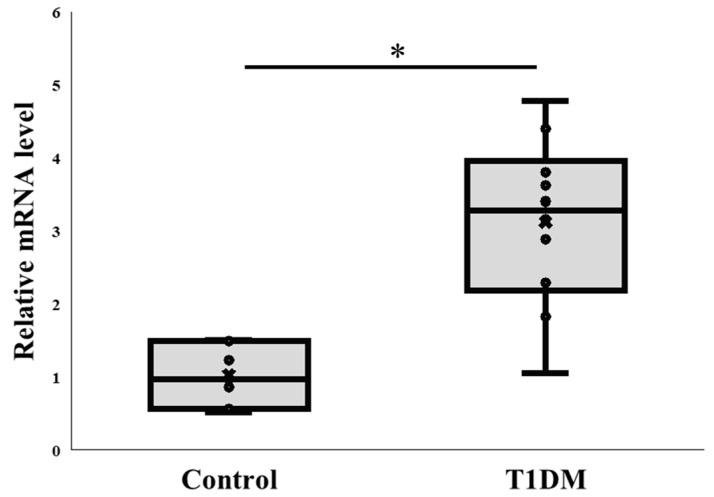
PAD4 mRNA levels in the pancreas. Comparison of PAD4 mRNA expression between control and T1DM groups. * *p* < 0.05. Results are presented as mean ± S.E.M. (Control, n = 7; T1DM, n = 10).

PAD4 is implicated in NETs and plays a significant role in NETosis. Thus, we investigated the pattern of PAD4 gene expression in pancreatic tissue from STZ-treated and untreated control rats concurrently with the protein level investigations. According to our RT-PCR results, pancreatic samples from treated animals had a noticeably greater PAD4 mRNA level than the controls. (2^−ΔΔCT^: 1.018 ± 0.408 vs. 3.122 ± 1.151) (Figure 2). Neutrophil infiltration may be explained by the elevated level of PAD4 mRNA, but more research is required to validate this.

### 2.4. The Expression of PAD4 and Citrullinated Protein in the Pancreas at the Sixth Week in Exp A

**Figure 3:**
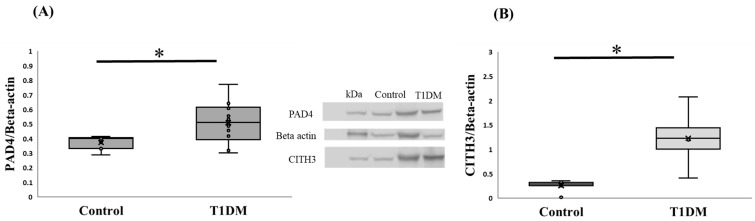
(**A**) PAD4 protein expression in the pancreas. PAD4 protein level between control and T1DM groups against internal control (Beta-actin); PAD4 expression in the T1DM group was greater than in the control in the pancreas (* *p* < 0.05). Results are presented as mean ± S.E.M. (Control, n = 7; T1DM, n = 10) (**B**) CITH3 levels in the pancreas. Change in the expression of citrullinated histone-3 (CITH3) between control and T1DM groups. The result indicated the successful induction of T1DM according to the citrullinated level (* *p* < 0.05). Results are presented as mean ± S.E.M. (Control, n = 8; T1DM, n = 6).

Our primary findings on PAD4 are significant enough to meaningfully depict the difference between control and T1DM groups in the pancreas in order to determine NET (*p* < 0.05). Figure 3 shows that the T1DM group has higher levels of expressed PAD4 than the control group, as determined by quantifying the protein expression and normalizing it to the internal control (beta actin) from extracted pancreas. According to a review paper, PADs in various tissues may play a significant role as a regulator during the pathogenic process of type 1 diabetes [6]. Additionally, we compared the levels of CITH3 in the control and T1DM groups and found that the latter had a considerably higher level of CITH3 than the former. To guarantee the citrullinated substrate for PAD4 and assess the effectiveness of the disease animal model, we assessed the intensity of CITH3 in the pancreas between the control and T1DM groups in the current investigation, which was based on the hypothesis of post-translational modification (Figure 3).

### 2.5. Ca^2+^: A Prerequisite Cofactor for Citrullination Among PADs in the Sixth Week in Exp A

**Figure 4:**
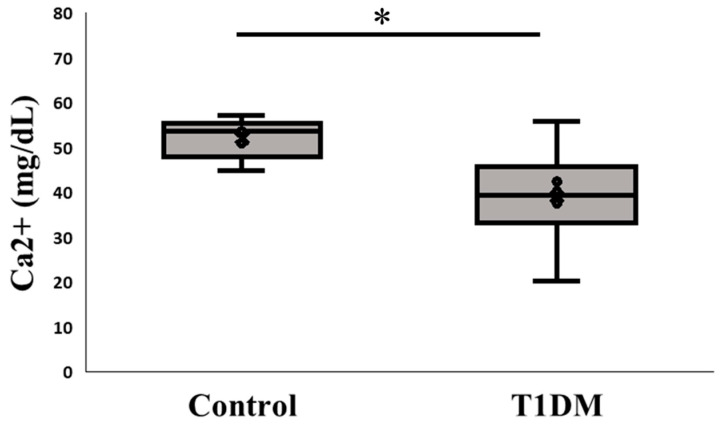
Ca^2+^ content in the pancreas. Change in the expression of calcium ion between control and T1DM groups; Ca^2+^ is a cofactor for PAD4 and our measurement presents a profound impact on the pancreas between control and T1DM groups (* *p* < 0.05). Results are presented as mean ± S.E.M. (Control, n = 5; T1DM, n = 6).

Because PAD4 is a calcium-dependent enzyme, when calcium becomes stuck in the active pocket, it starts to citrullinate [10]. Here, we examined the alteration in the pancreatic microenvironment linked to PAD4 expression by measuring Ca^2+^ levels between the control and T1DM groups. According to our findings, PAD4 may bind to calcium ions and boost citrullination activity in T1DM, causing the pancreas to produce less calcium ions than the control group (Figure 4).

### 2.6. Blood Glucose Measurement Before Termination in Exp A and B

**Figure 5:**
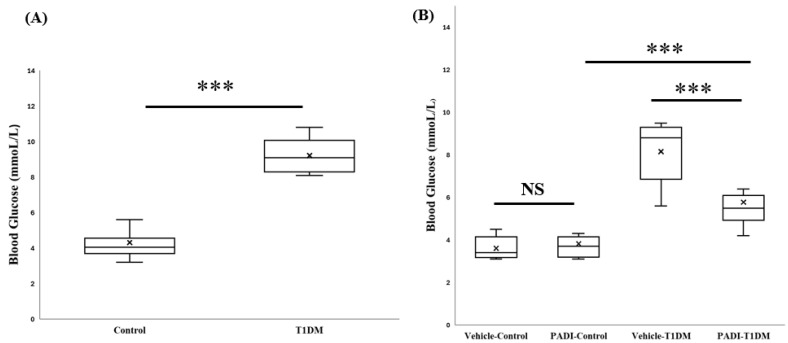
Blood glucose level before termination. (**A**) In the 6th week, by confirming blood glucose levels, T1DM was successfully induced. (**B**) In experiment B, we used a PAD inhibitor (PADI) to further examine PAD4 expression. After ten days of dosing, we also checked the blood glucose level. *** *p* < 0.001 (all groups, n = 10).

In order to accurately determine the state of TDM, the procedure for STZ-induced T1DM induction [16] requires blood glucose to be confirmed prior to sacrifice [16]. In experiments A and B, before termination, we measured the blood glucose levels. Every blood glucose reading between the STZ and control groups was significant (Figure 5).

### 2.7. The Expression of PAD4 mRNA in the Pancreas in Exp B

**Figure 6:**
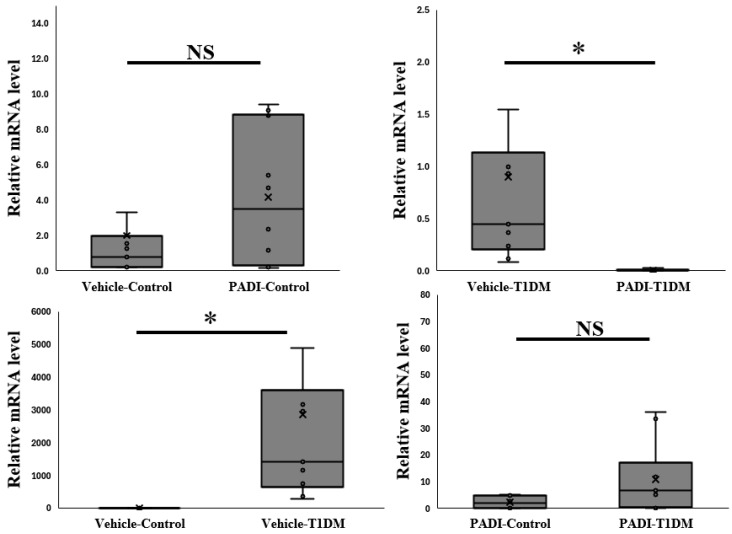
The expression of PAD4 mRNA level in the pancreas in exp B. The relative mRNA level is significant between vehicle–T1DM and PADI-T1DM (* *p* < 0.05, all groups n = 10).

In order to determine whether rats can be used to measure PAD4 in the same way as mice, we kept inhibiting PADs. We gave rats IP injections in the sixth week for ten days over consecutive days (5 mg/kg/day). According to our data, rats are another suitable animal model for researching the expression of PAD4 (Figure 6).

### 2.8. NETosis Level in the Serum and Pancreas in Exp B

**Figure 7:**
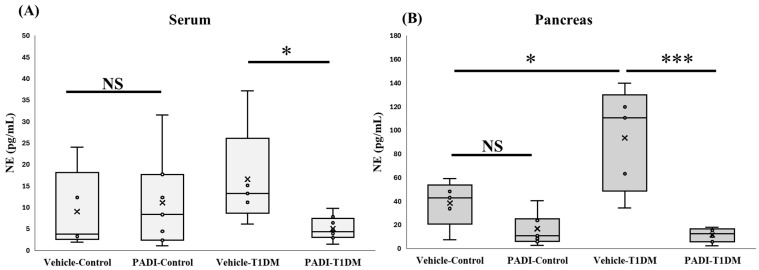
NETosis level in the serum and pancreas in exp B. A component of extracellular trap formation is neutrophil elastase (NE). We employed Cl-amidine, one of the PAD inhibitors (PADI). * *p* < 0.05, *** *p* < 0.001, NS: non-significant (**A**) vehicle–control, n = 5; PADI–control, n = 7; vehicle–TIDM, n = 5; PADI-T1DM, n = 8; (**B**) vehicle–control, n = 5; PADI-control, n = 7; vehicle–TIDM, n = 5; PADI-T1DM, n = 7.

Neutrophil elastase (NE) plays a critical role in extracellular trap formation (NETosis). We looked at NE expression in the pancreas and serum to make sure T1DM was successful in causing NETosis. Despite not being evident in the serum, Cl-amidine suggested a substantial difference between the control and STZ groups (Figure 7).

## 3. Discussion

The role of citrullination and peptidylarginine deiminases (PADs) in T1DM has recently gained significant attention. Most previous studies have concentrated on PAD2 and PAD4, particularly elucidating the mechanisms of glucokinase citrullination in the pancreas and liver of NOD mice [23,24]. In contrast, the present study specifically investigated PAD4 expression and its link with inflammation in an STZ-induced T1DM rat model (Table 2).

Various PTMs, including citrullination, oxidation, phosphorylation, acetylation, methylation, deamidation, and carbonylation, have been implicated in modulating T1DM autoimmunity, glucose regulation, and insulin metabolism. However, whether these PTMs are causative factors or secondary consequences of T1DM pathology remains uncertain [25]. Although animal models and ex vivo studies have provided valuable insights, their findings cannot fully replicate the complexity of human T1DM. Previous research demonstrated an increase in citrullinated protein levels in STZ-induced T1DM models [6]. However, the specific contribution of PAD4 to disease pathogenesis requires further clarification.

PAD4 is a critical mediator of NETosis, a process exacerbating inflammatory responses. While numerous studies have reported elevated citrullinated protein levels in autoimmune diseases, the direct evaluation of PAD4 protein expression in T1DM remains limited. Our results demonstrate significantly higher PAD4 mRNA and protein levels in the T1DM group compared to the controls (Figure 2 and Figure 3). This observation aligns with the findings from various immune-mediated conditions, where increased PAD activity correlates with disease severity. Moreover, PAD4 activation is calcium-dependent, and our data reveal decreased pancreatic Ca^2+^ content in the T1DM group (Figure 4), suggesting calcium mobilization into PAD4′s active site during enzymatic activation. Simultaneously, increased levels of CITH3, a hallmark biomarker of NETosis, were observed, further reinforcing PAD4′s role in disease progression. Recent studies highlighted that PAD4 can initiate NETosis through enhanced reactive oxygen species (ROS) production via NADPH oxidase and mitochondrial dysfunction [12]. Although reduced circulating neutrophil numbers have been noted in prediabetes and early T1DM [12,26], our findings underscore enhanced local pancreatic NETosis activity rather than systemic changes. Supporting this, the results emphasize that PAD4-driven NETosis substantially contributes to autoimmune disease pathogenesis, including T1DM, and that PAD4 inhibition represents a promising therapeutic strategy [13,27].

Moreover, while a previous cross-sectional study associated circulating PAD4 gene expression with neutrophil counts in long-term T1DM patients [28], our findings indicate that pancreatic PAD4 mRNA levels may not directly correlate with neutrophil numbers, suggesting a need for further investigation into localized immune responses (Figure 6). We also analyzed several pro-inflammatory cytokines, including IL-6, IL-18, IL-33, TNF-α, and IFN-γ, which are predominantly secreted by macrophages and T cells during insulitis, leading to β-cell apoptosis and enhanced neutrophil recruitment [29,30,31,32]. Our data corroborate the previous observations, demonstrating increased cytokine levels correlating with β-cell mass reduction. Conversely, the anti-inflammatory cytokine IL-10, which provides protection against β-cell damage, was decreased in T1DM, consistent with the earlier findings [33]. Collectively, our data support the hypothesis that PAD4-mediated NETosis contributes significantly to pancreatic inflammation and β-cell destruction in T1DM. The pharmacological inhibition of PAD4 has been shown to attenuate disease progression in animal models (Figure 7). Notably, Cl-amidine, a pan-PAD inhibitor, has demonstrated protective effects against diabetes development in NOD mice by irreversibly inactivating PAD enzymes [34]. The promising results reported further strengthen the potential for PAD4 inhibitors as therapeutic agents in managing T1DM and other autoimmune diseases [7,35].

While Cl-amidine and PAD4 have been extensively studied in mouse models of autoimmune diabetes, including NOD mice [34], our study provides new evidence supporting the utility of rats as an alternative preclinical model. Table 2 summarizes the key differences between the models. Mice are more sensitive to STZ toxicity, especially at high doses (200 mg/kg), and may exhibit higher mortality, limiting the longitudinal studies. In contrast, Wistar rats tolerate STZ better (e.g., 60 mg/kg i.p. in our protocol) and allow for stable disease induction and therapeutic intervention with lower variability.

PAD4 expression has been previously implicated in NETosis and β-cell damage in mouse models [6], but limited data are available on PAD4’s role in the pancreas of STZ-induced rats. Our findings demonstrate that PAD4 mRNA and protein levels are significantly elevated in diabetic rat pancreas, paralleling the increases in CITH3 and neutrophil elastase activity. These effects were mitigated by Cl-amidine treatment, confirming functional inhibition. Therefore, our rat model not only reproduces key inflammatory features observed in mouse models, but also offers higher tissue yield and a better pharmacokinetic platform for therapeutic testing, as previously noted by Furman [11].

Thus, the rat model used in this study represents a robust and physiologically relevant system to study PAD4-driven NETosis in T1DM. It complements the mouse data by providing translational insights with reduced mortality, reproducible β-cell destruction, and the ability to quantify molecular changes in the pancreas at both mRNA and protein levels.

In conclusion, our study underscores the critical involvement of PAD4 in STZ-induced T1DM, suggesting that enhanced PAD4 expression, decreased calcium levels due to enzyme activation, and elevated pro-inflammatory cytokines collectively contribute to disease pathogenesis. Future research focusing on upstream regulatory mechanisms, including epigenetic modifications, such as histone methylation and DNA/RNA methylation, may provide deeper insights into PAD4′s role in T1DM and inform new therapeutic strategies.

### Limitations of This Study

First, the sample size, while adequate for detecting consistent molecular trends across key endpoints, may limit the statistical power for broader generalizations. Nevertheless, the use of multiple complementary assays (qPCR, Western blot, ELISA, calcium measurement, and immunohistochemistry) provides converging evidence supporting the reliability of our findings. Second, this study employed a single animal model (STZ-induced T1DM in rats), which, although well-established and physiologically relevant, may not capture the full spectrum of immune mechanisms involved in human T1DM. Third, we focused on one PAD inhibitor, Cl-amidine, without comparing alternative compounds or PAD4-specific genetic approaches.

Finally, our analysis was limited to pancreatic tissue; future studies incorporating systemic assessments and longitudinal designs will be valuable to further clarify the role of PAD4 in diabetes pathophysiology. Despite these limitations, our study offers novel insights into PAD4-mediated NETosis and inflammation in the diabetic pancreas and provides a strong rationale for continued translational research in this area.

## 4. Materials and Methods

Male Wistar rats from BRC in Szeged, Hungary, were used in the experiment. They were kept in cages. Before they were sacrificed, the habitat was maintained for six weeks at a temperature in the range of 20–22 °C and a humidity level in the range of 40–50% with a 12 h cycle of light and dark. The rats, which weighed between 200 and 230 g, were given unlimited access to food and water. At the start and finish of the trial, weighted body weight measures were made. To conduct this experiment, we needed the bare minimum of rats, and the resource equation from our last trial served as a guide to determine the necessary sample size. This study was also piloted in our lab. Each procedure complied with the European Parliament’s Directive (2010/63EU) and was authorized by European Community regulations. (University of Szeged ethics license: XXXIX./2040/2023).

### 4.1. Experimental Protocol

**Figure 8:**
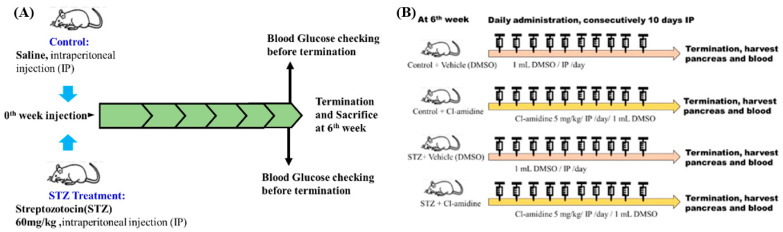
Experiment A and B designs (exp A and exp B). (**A**) Verification that the STZ dosage of 60 mg/kg was feasible for creating diabetic rats. (**B**) Cl-amidine, a PAD inhibitor, is frequently used to study PAD4 expression in mice. Here, we also evaluated the expression of PAD4 in rats by using Cl-amidine.

After acclimation, Wistar Rats were able to discriminate between the T1DM group and the control group. The T1DM group received an intraperitoneal (IP) injection of streptozotocin (STZ) at a dose of 60 mg/kg/body weight (dissolved in saline) [11,36], while the control group received an IP injection of saline in exp A. and B (Figure 8). First, we performed experiment A to confirm PAD4 expression in STZ-induced diabetic rats (diabetes induced for 6 weeks). Second, we conducted a separate PAD4 inhibition trial (exp B) to evaluate the role of PAD4 in STZ-induced diabetic rats. (Diabetes-induced duration with PAD4 inhibition: 6 weeks + 10 days of Cl-amidine IP).

In experiment B, we inhibited PAD4. The dose of Cl-amidine (Merck, 506282, Rahway, NJ, USA) was 5 mg/kg/IP for ten consecutive days. The vehicle for dissolving Cl-amidine was dimethyl sulfoxide (DMSO, Merck, 102952, Rahway, NJ, USA), which was treated in the control group. Throughout the trial, each rat’s weight was noted, and at the sixth week, it was euthanized. Before termination, each rat fasted for a day to allow for blood glucose measurements. Each rat’s pancreas, blood, and liver were meticulously removed and used. Every tissue was stored in Triton at −80 °C for biological measurements.

### 4.2. Measurement of Calcium Ion in the Pancreas in Exp A

The Calcium Detection Assay Kit (Abcam, ab102505, Waltham, MA, USA, range: 0.4–100 mg/dL) was used to measure the Ca^2+^ level after pancreas resection. All the pancreases were homogenized in PBS plus NP-40, then put on ice and centrifuged for five minutes at 10,000 rpm. The supernatants’ measurements were carried out using the specified standards. Optical densities (ODs) were measured at λ = 575 nm. The data are expressed in nanograms per well.

### 4.3. Western Blot of CITH3, PAD4 in Exp A

The pancreatic samples were stored in a solution that contained RIPA buffer (Merck Millipore, Burlington, MA, USA) and 1/10 of the final volume of phenylmethylsulfonyl fluoride (PMSF) (Sigma-Aldrich, Budapest, Hungary). Using the UP-100H Ultrasonic Homogenizer (Hielscher Ultrasonics, Teltow, Germany), the homogenates were homogenized three times for 10 s on ice before being centrifuged at 15,000× *g* for 10 min at 4 °C. The protein content of the supernatants was determined using the Bradford test. A total 50 µg of protein was extracted from each sample according to its protein content and placed onto 10% sodium dodecyl sulfate (SDS) polyacrylamide gels for two hours at a voltage of 90 V. The gels were transferred to nitrocellulose membranes after 2.5 h at 35 V. The membranes were dyed with Ponceau S and then washed with TBS-T (pH 7.4). The membranes were blocked in 5% BSA throughout the night. After blocking, blots were washed three times for 10 min in TBS-T. The first pair of antibodies—anti-PAD4 (Dilution: 1:3000, Catalog Number: 17373-1-AP, Proteintech, Manchester, UK) and anti-CITH3 (1:750, Catalog Number: ab5103, citrulline R2&R8&R17, Abcam, Cambridge, UK)—were incubated for two hours at room temperature. Then, using anti-mouse antibodies conjugated with horseradish peroxidase (DAKO Agilent, Santa Clara, CA, USA), they were kept for 1 h at room temperature. Quantity One Software version 4.5 (Bio-Rad Laboratories, Hercules, CA, USA) was utilized for analysis, and an enhanced chemiluminescence system (ECL Plus, Amersham Pharmacia Biotech., Buckinghamshire, UK) was used to display band pictures. Each membrane was stripped and used for the detection of β-actin for normalization (ab20272, Abcam, Cambridge, UK; anti-mouse secondary antibody, DAKO Agilent, Santa Clara, CA, USA). The results were normalized to β-actin and presented as relative expressions.

### 4.4. Measurement of Pancreatic TNF-α, IFN-γ, IL-6, IL-10, IL-18, NETosis, and IL-33 Concentrations in Exp A

After sacrifice, TNF-α (Invitrogen, Catalog: BMS622), IFN-γ (Invitrogen, Catalog: BMS621), IL-6, IL-10 (Invitrogen, Catalog: BMS625), IL-18 (Invitrogen, Catalog: KRC2341), NETosis (ELK Biotechnology, Catalog: ELK1521), and IL-33 (Abcam, Catalog: ab236714) levels were measured using ELISA kits obtained from several vendors and used in compliance with each manufacturer’s recommendations.

### 4.5. RNA Extraction, Reverse Transcription, and Quantitative Real-Time Polymerase Chain Reaction (qRT-PCR)

Pancreatic samples were homogenized in 1 milliliter of TRIzol reagent (Life Technologies, Carlsbad, CA, USA) using the MT-13K-L small, portable, homogenizer (MiuLab, Yuhang District, Hangzhou, China). Following the manufacturer’s recommendations, the samples were centrifuged for 10 min at 13,000 rpm, and total RNA was isolated from the upper phase using the Monarch Total RNA Miniprep Kit (BioLabs, Los Angeles, CA, USA). The quantity and quality of isolated RNA were evaluated using DeNovix DS-11 (DeNovix, Wilmington, DE, USA). The QuantiTect reverse transcription kit (Qiagen, Hilden, Germany) was used to convert 100 ng of total RNA to cDNA in accordance with the manufacturer’s instructions. cDNA levels were determined using QPCR and the Rotor-Gene Q real-time PCR System (Qiagen). The QuantiNova SYBR Green PCR Kit (Qiagen) was used to perform reactions using the following primer sets:

PAD4 sense: GCTCCCTCTCATCAGTTCCA.
PAD4 antisense: GGCTTGTCACTCGAGTTTTGA.
HPRT sense: CATTAATATTTAACGATGTGGATGCGTTTCA.
HPRT antisense: GCCTACCATCTTTAAACTGCACAAT.

In order to avoid false-positive results from the amplification of contaminated genomic DNA during the cDNA synthesis, we selected primers that had spanexon–exon junctions. There were at least four biological replicates used for each measurement. The ratio of each PAD4 mRNA to the HPRT was calculated using the 2^−ΔΔCT^ technique [37].

### 4.6. Statistical Analysis

The results are reported as mean ± SEM for per group. For statistical comparisons, due to the normal distribution of all group data, the student’s two-tailed *t*-test was used. *p* values less than 0.05 were considered significant differences.

## 5. Conclusions

This study provides compelling evidence that PAD4 overexpression and activity are closely associated with pancreatic inflammation and β-cell damage in STZ-induced T1DM. Increased PAD4 mRNA and protein levels, elevated citrullinated histone H3 expression, and decreased pancreatic calcium content collectively point to enhanced PAD4-mediated NETosis as a key contributor to disease pathogenesis. Additionally, the observed upregulation of pro-inflammatory cytokines and downregulation of IL-10 further support a pro-inflammatory microenvironment promoting β-cell destruction.

The findings align with the recent evidence suggesting that PAD4-driven NET formation exacerbates autoimmune responses and highlight PAD4 as a potential therapeutic target in T1DM. PAD inhibitors, such as Cl-amidine, have shown promise in preclinical models by mitigating NETosis and preserving β-cell function.

Future studies should aim to elucidate the upstream signaling pathways regulating PAD4 activation, explore the interplay between PAD4 and other PTMs, and investigate the potential of PAD4 inhibition in clinical settings. Targeting PAD4-mediated NETosis may represent a novel and effective strategy to prevent or slow the progression of T1DM.

## Figures and Tables

**Table 1:** Concentration of various inflammatory and anti-inflammatory cytokines in the pancreas. Summary of the analysis of various proinflammatory/anti-inflammatory cytokines in the pancreas of STZ-treated diabetic rats and control. * *p* ≤ 0.05, n = 10 (unit: pg/mg protein).

Group	CXCL-1	IFN-γ	IL-6	IL-18	IL-33	IL-10
Control	33.02 ±3.34	21.62 ± 2.44	40.81 ± 5.63	77.67 ± 4.83	708.38 ± 76.19	451.95 ± 152.31 *
STZ	46.85 ± 3.32 *	29.46 ± 2.00 *	55.44 ± 3.29 *	130.75 ± 17.46 *	1027.26 ± 113.47 *	153.71 ± 18.73
Cytokine	inflammatory	inflammatory	inflammatory	inflammatory	inflammatory	anti-inflammatory

**Table 2:** A comparison of rats and mice treated with STZ and Cl-amidine.

	Rats (Weight: 180~200 g)	Mice (Weight: ~25 g)	Note
Strains	Sprague Dawley, Wistar	CD-1, C57BL-6	Male mice or rats are more susceptible to STZ than females
STZ-induced protocol and STZ dose	Single STZ dose: 40~70 mg/kg	1. Low STZ dose: 40~50 mg/kg/day, 5 consecutive day 2. Single, high STZ dose: 200 mg/kg	Caution: Mice that undergo STZ-induced experiments are more likely to die (Figure 1)
Cl-amidine dose	In the current trial, we administered 5 mg/kg/day via IP injection for ten days in a row	Oral gavage or IP: 5~75 mg/kg/day for 30 consecutive days in accordance with other articles	Caution: rats are heavier than mice, hence more Cl-amidine will be consumed by them during the experiment Consequently, the cost is increasing
PAD4 expression and the capacity of the inhibition of Cl-amidine	In the current work, we confirmed that PAD4 expression and Cl-amidine inhibition are significant between the control and STZ-T1DM groups	To date, the majority of the research has used mice to treat Cl-amidine and study any disorders connected to PAD4 expression	Some high STZ-sensitive strains may treat low-dose Cl-amidine

## Data Availability

Data will be made available upon reasonable request to the coauthors.

## References

[1] György B., Tóth E., Tarcsa E., Falus A., Buzás E.I. (2006). Citrullination: A Posttranslational Modification in Health and Disease. Int. J. Biochem. Cell Biol..

[2] Ramazi S., Zahiri J. (2021). Post-Translational Modifications in Proteins: Resources, Tools and Prediction Methods. Database.

[3] McLaughlin R.J., Spindler M.P., Van Lummel M., Roep B.O. (2016). Where, How, and When: Positioning Posttranslational Modification Within Type 1 Diabetes Pathogenesis. Curr. Diab. Rep..

[4] Mauracher L.-M., Posch F., Martinod K., Grilz E., Däullary T., Hell L., Brostjan C., Zielinski C., Ay C., Wagner D.D. (2018). Citrullinated Histone H3, a Biomarker of Neutrophil Extracellular Trap Formation, Predicts the Risk of Venous Thromboembolism in Cancer Patients. J. Thromb. Haemost..

[5] Guo G., Liu Z., Yu J., You Y., Li M., Wang B., Tang J., Han P., Wu J., Shen H. (2024). Neutrophil Function Conversion Driven by Immune Switchpoint Regulator against Diabetes-Related Biofilm Infections. Adv. Mater..

[6] Wong S.L., Demers M., Martinod K., Gallant M., Wang Y., Goldfine A.B., Kahn C.R., Wagner D.D. (2015). Diabetes Primes Neutrophils to Undergo NETosis, Which Impairs Wound Healing. Nat. Med..

[7] Yang M.-L., Sodré F.M.C., Mamula M.J., Overbergh L. (2021). Citrullination and PAD Enzyme Biology in Type 1 Diabetes—Regulators of Inflammation, Autoimmunity, and Pathology. Front. Immunol..

[8] Shen Y., You Q., Wu Y., Wu J. (2022). Inhibition of PAD4-Mediated NET Formation by Cl-Amidine Prevents Diabetes Development in Nonobese Diabetic Mice. Eur. J. Pharmacol..

[9] Yang C., Dong Z.-Z., Zhang J., Teng D., Luo X., Li D., Zhou Y. (2021). Peptidylarginine Deiminases 4 as a Promising Target in Drug Discovery. Eur. J. Med. Chem..

[10] He W., Xi Q., Cui H., Zhang P., Huang R., Wang T., Wang D. (2022). Forsythiaside B Ameliorates Coagulopathies in a Rat Model of Sepsis through Inhibition of the Formation of PAD4-Dependent Neutrophil Extracellular Traps. Front. Pharmacol..

[11] Streptozotocin-Induced Diabetic Models in Mice and Rats-Furman-2021-Current Protocols-Wiley Online Library. https://currentprotocols.onlinelibrary.wiley.com/doi/full/10.1002/cpz1.78.

[12] Zhu Y., Xia X., He Q., Xiao Q.-A., Wang D., Huang M., Zhang X. (2023). Diabetes-Associated Neutrophil NETosis: Pathogenesis and Interventional Target of Diabetic Complications. Front. Endocrinol..

[13] Zhu D., Song W., Jiang Z., Zhou H., Wang S. (2022). Citrullination: A Modification Important in the Pathogenesis of Autoimmune Diseases. Clin. Immunol..

[14] Kraaij T., Tengström F.C., Kamerling S.W.A., Pusey C.D., Scherer H.U., Toes R.E.M., Rabelink T.J., van Kooten C., Teng Y.K.O. (2016). A Novel Method for High-Throughput Detection and Quantification of Neutrophil Extracellular Traps Reveals ROS-Independent NET Release with Immune Complexes. Autoimmun. Rev..

[15] Chrysanthopoulou A., Mitroulis I., Apostolidou E., Arelaki S., Mikroulis D., Konstantinidis T., Sivridis E., Koffa M., Giatromanolaki A., Boumpas D.T. (2014). Neutrophil Extracellular Traps Promote Differentiation and Function of Fibroblasts. J. Pathol..

[16] Öğüten P.N., Öztürk S.E., Dikmen M. (2024). The Investigation of Cytotoxic and Apoptotic Activity of Cl-Amidine on the Human U-87 MG Glioma Cell Line. Medicine.

[17] Yao H., Cao G., Liu Z., Zhao Y., Yan Z., Wang S., Wang Y., Guo Z., Wang Y. (2022). Inhibition of Netosis with PAD Inhibitor Attenuates Endotoxin Shock Induced Systemic Inflammation. Int. J. Mol. Sci..

[18] Du J., Wang N., Sun H., Zheng L., Qi X. (2023). Cl-Amidine Attenuates Lipopolysaccharide-Induced Inflammation in Human Gingival Fibroblasts via the JNK/MAPK, NF-κB, and Nrf2 Signalling Pathways. Hum. Cell.

[19] Long X., Yuan Q., Tian R., Zhang W., Liu L., Yang M., Yuan X., Deng Z., Li Q., Sun R. (2024). Efficient Healing of Diabetic Wounds by MSC-EV-7A Composite Hydrogel via Suppression of Inflammation and Enhancement of Angiogenesis. Biomater. Sci..

[20] Yang S., Wang S., Chen L., Wang Z., Chen J., Ni Q., Guo X., Zhang L., Xue G. (2023). Neutrophil Extracellular Traps Delay Diabetic Wound Healing by Inducing Endothelial-to-Mesenchymal Transition via the Hippo Pathway. Int. J. Biol. Sci..

[21] Altamura S., Lombardi F., Palumbo P., Cinque B., Ferri C., Del Pinto R., Pietropaoli D. (2024). The Evolving Role of Neutrophils and Neutrophil Extracellular Traps (NETs) in Obesity and Related Diseases: Recent Insights and Advances. Int. J. Mol. Sci..

[22] Sun B., Chen H., Xue J., Li P., Fu X. (2023). The Role of GLUT2 in Glucose Metabolism in Multiple Organs and Tissues. Mol. Biol. Rep..

[23] Mondal S., Thompson P.R. (2019). Protein Arginine Deiminases (PADs): Biochemistry and Chemical Biology of Protein Citrullination. Acc. Chem. Res..

[24] Zhou Y., Chen B., Mittereder N., Chaerkady R., Strain M., An L.-L., Rahman S., Ma W., Low C.P., Chan D. (2017). Spontaneous Secretion of the Citrullination Enzyme PAD2 and Cell Surface Exposure of PAD4 by Neutrophils. Front. Immunol..

[25] Sharma C., Hamza A., Boyle E., Donu D., Cen Y. (2024). Post-Translational Modifications and Diabetes. Biomolecules.

[26] Battaglia M., Petrelli A., Vecchio F. (2019). Neutrophils and Type 1 Diabetes: Current Knowledge and Suggested Future Directions. Curr. Opin. Endocrinol. Diabetes Obes..

[27] Ciesielski O., Biesiekierska M., Panthu B., Soszyński M., Pirola L., Balcerczyk A. (2022). Citrullination in the Pathology of Inflammatory and Autoimmune Disorders: Recent Advances and Future Perspectives. Cell. Mol. Life Sci..

[28] Aukrust S.G., Holte K.B., Opstad T.B., Seljeflot I., Berg T.J., Helseth R. (2022). NETosis in Long-Term Type 1 Diabetes Mellitus and Its Link to Coronary Artery Disease. Front. Immunol..

[29] Ramadan J.W., Steiner S.R., O’Neill C.M., Nunemaker C.S. (2011). The Central Role of Calcium in the Effects of Cytokines on Beta-Cell Function: Implications for Type 1 and Type 2 Diabetes. Cell Calcium.

[30] Garley M., Jabłońska E., Surażyński A., Grubczak K., Ratajczak-Wrona W., Iwaniuk A., Dąbrowska D., Pałka J.A., Moniuszko M. (2017). Original Article Cytokine Network & NETs. Folia Biol..

[31] Heinrich P.C., Behrmann I., Haan S., Hermanns H.M., Müller-Newen G., Schaper F. (2003). Principles of Interleukin (IL)-6-Type Cytokine Signalling and Its Regulation. Biochem. J..

[32] Xiao J., Li J., Cai L., Chakrabarti S., Li X. (2014). Cytokines and Diabetes Research. J. Diabetes Res..

[33] Glowacka E., Banasik M., Lewkowicz P., Tchorzewski H. (2002). The Effect of LPS on Neutrophils from Patients with High Risk of Type 1 Diabetes Mellitus in Relation to IL-8, IL-10 and IL-12 Production and Apoptosis In Vitro: Polymorphonuclear Neutrophils Apoptosis in DM1. Scand. J. Immunol..

[34] Sodré F.M.C., Bissenova S., Bruggeman Y., Tilvawala R., Cook D.P., Berthault C., Mondal S., Callebaut A., You S., Scharfmann R. (2021). Peptidylarginine Deiminase Inhibition Prevents Diabetes Development in NOD Mice. Diabetes.

[35] Abacar K., Macleod T., Direskeneli H., McGonagle D. (2024). How Underappreciated Autoinflammatory (Innate Immunity) Mechanisms Dominate Disparate Autoimmune Disorders. Front. Immunol..

[36] Ghasemi A., Jeddi S. (2023). Streptozotocin as a Tool for Induction of Rat Models of Diabetes: A Practical Guide. Excli J..

[37] Melo Z., Gutierrez-Mercado Y.K., Garcia-Martínez D., Portilla-de-Buen E., Canales-Aguirre A.A., Gonzalez-Gonzalez R., Franco-Acevedo A., Palomino J., Echavarria R. (2020). Sex-Dependent Mechanisms Involved in Renal Tolerance to Ischemia-Reperfusion: Role of Inflammation and Histone H3 Citrullination. Transpl. Immunol..

